# Seeking validation in the digital age: The impact of validation seeking on self-image and internalized stigma among self- vs. clinically diagnosed individuals on r/ADHD

**DOI:** 10.1371/journal.pone.0331856

**Published:** 2025-10-01

**Authors:** Xinyu Zhang, Yoo Jung Oh, Yunhan Zhang, Jianfeng Zhu

**Affiliations:** 1 Department of Communication, Michigan State University, East Lansing, Michigan, United States of America; 2 School of History and Culture, Department of Archival Science, Hubei University, Wuhan, Hubei, China; 3 Department of Computer Science, Kent State University, Kent, Ohio, United States of America; UTM Skudai: Universiti Teknologi Malaysia, MALAYSIA

## Abstract

The digital age has fueled a surge in ADHD self-diagnosis as people turn to online platforms for mental health information. However, the relationship between validation-seeking behaviors and self-perception in these online communities and users’ self-perception has received limited scholarly focus. Drawing on self-verification theory and utilizing natural language processing to analyze 452,026 posts from the r/ADHD subreddit, our study uncovers distinct patterns in validation-seeking behaviors. Results show that (a) self-diagnosed individuals with ADHD are more likely to seek social validation and media validation and to report higher levels of negative self-image and internalized stigma than clinically diagnosed individuals, (b) social validation was strongly associated with both positive and negative self-perceptions; and (c) diagnosis status significantly moderated these relationships, such that the effects of social validation on self-image and stigma were consistently weaker for the self-diagnosed group. Theoretically, this study extends self-verification theory by demonstrating that professional verification hierarchically moderates self-verification effectiveness. This implies a practical need for clinicians to acknowledge online validation seeking and for digital communities to affirm user experiences while mitigating stigma.

## Introduction

The identification of Attention-Deficit/Hyperactivity Disorder (ADHD) has traditionally been anchored in clinical diagnosis, a process reliant on professional assessment. However, this established pathway is fraught with significant barriers that limit its accessibility. Systemic challenges, including prolonged wait times for specialists, prohibitive costs, and geographic disparities in healthcare, create substantial obstacles for many individuals [1]. Compounding these structural issues is the persistent social stigma associated with seeking a formal psychiatric diagnosis. Consequently, a growing number of individuals are driven to seek alternative avenues for understanding their experiences, moving beyond the confines of traditional healthcare systems.

This search for understanding increasingly occurs within complex socio-technical environments, particularly social media platforms like TikTok and Reddit. The architectural affordances of these platforms, such as algorithmically curated content streams and large-scale, interactive community forums, fundamentally shape how users encounter and interpret health information [2]. This technological infrastructure fosters a participatory health culture, where individuals transition from passive recipients of medical knowledge to active agents in constructing and validating health-related identities [Foster and Ellis, 2024] [3,4]. As a result, these digital spaces have become primary arenas for self-exploration and community formation around conditions like ADHD.

The emergence of these online communities presents a significant duality. On one hand, they offer invaluable benefits, providing spaces for peer support, reducing feelings of isolation, and empowering individuals through frameworks like the neurodiversity movement, which reframes neurological differences as natural variations [5,6]. On the other hand, the same unregulated and user-driven dynamics that foster community also facilitate the rapid spread of misinformation, the oversimplification of complex clinical traits, and the promotion of unverified advice [7]. This creates a complex information environment where the potential for relief coexists with the risk of diagnostic confusion.

While existing research has documented these dual outcomes, the psychological mechanisms governing them remain largely underexplored. Central to this dynamic is the process of validation, the social affirmation of an individual’s experiences and identity. This pursuit of affirmation is well-explained by Self-Verification Theory [8], which posits a fundamental drive to seek confirmation for one’s existing self-concept. Consequently, the act of receiving validation becomes a crucial process of identity consolidation. A significant gap therefore exists in understanding how the source of this validation, whether from online peers or a clinical professional, differentially impacts individuals according to their diagnostic status [9].

This study seeks to address these gaps by analyzing validation-seeking behaviors among self-diagnosed and clinically diagnosed individuals in the r/ADHD Reddit community. Using natural language processing, we examine 452,026 posts over 14 years, we examine the relationship between diagnosis status, validation-seeking, and psychological outcomes. The goal is to elucidate how different forms of validation influence expressions of self-perception and internalized stigma, and whether an individual’s diagnosis status (self-diagnosed versus clinically diagnosed) moderates these critical relationships.

## Literature review

### ADHD and medical industrial complex

The pharmaceutical industry’s substantial financial investment in ADHD research has created a complex relationship between commercial interests and scientific legitimacy. The global ADHD medication market, valued at $15.8 billion in 2023 and projected to reach $24.6 billion by 2032, extends its influence through funding clinical trials, medical education programs, and patient advocacy organizations [10]. Systematic reviews demonstrate that pharmaceutical sponsorship consistently produces results more favorable to sponsors compared to independent research, even when following rigorous protocols [11]. This pattern has contributed to widespread public skepticism about ADHD’s legitimacy, with critics arguing that companies have vested interests in expanding diagnostic criteria and treatment populations [12]. Such erosion of trust in medical authority creates conditions where individuals seek alternative validation for their symptoms through non-professional sources.

The frequent revisions to ADHD diagnostic criteria have undermined confidence in the disorder’s scientific foundation. The DSM-5’s 2013 revision increased symptom onset age from 7 to 12 years and introduced adult-specific criteria, resulting in a 15% increase in adult diagnoses and suggesting that diagnostic boundaries reflect committee decisions rather than empirical discoveries [13,14]. The subjective nature of symptom assessment tools, combined with the absence of objective biomarkers, allows for considerable diagnostic variability between clinicians [15]. This instability erodes public trust because frequent changes in professional consensus suggest that ADHD exists on a continuum rather than as a discrete medical entity. Consequently, individuals question whether their symptoms meet evolving professional standards and seek validation through alternative channels.

These systemic failures in professional diagnosis have coincided with the rise of digital health platforms that offer alternative pathways for ADHD validation. Increasing adults now use the internet as their primary health information source, with ADHD-related content generating millions of views across social media platforms [16]. Social media algorithms create echo chambers where users encounter symptom descriptions that resonate with their experiences [17]. Online symptom checkers and self-assessment tools provide immediate feedback that reinforces diagnostic suspicions before professional evaluation occurs [18]. This digital landscape has created a parallel diagnostic culture where individuals validate their experiences through peer networks rather than medical professionals.

### Self-verification theory

Self-Verification Theory posits a fundamental motivation to maintain a stable and consistent self-concept, a drive that serves both epistemic needs for predictability and pragmatic goals for smoother social interactions [19]. Critically, this pursuit of confirmation extends to negative self-views. Research demonstrates that individuals with depression, for example, actively seek and prefer unfavorable feedback that aligns with their existing self-perceptions over positive, non-verifying assessments [8]. This established tendency to confirm even a negative self-concept provides a robust theoretical framework for examining the diagnostic and validation-seeking behaviors central to this study.

Within this theoretical framework, seeking a clinical diagnosis functions as a potent self-verification strategy. A formal diagnosis provides a structured, socially legitimized narrative that can resolve the cognitive dissonance caused by unexplained symptoms, thereby confirming an individual’s self-perceived identity [20]. When a diagnosis aligns with one’s self-concept, this verification can foster positive outcomes such as greater treatment engagement and improved well-being [21]. However, the drive for verification also presents significant risks. The diagnostic process may be skewed by confirmation bias, leading individuals to attend only to evidence supporting their preexisting beliefs [22], while the resulting label can foster a rigid self-view that may restrict personal growth and autonomy [23].

While a clinical diagnosis represents the formal pathway to self-verification, the digital age has fostered a parallel informal process: self-diagnosis. This alternative path is an active endeavor where individuals engage with specific digital ecosystems to construct and confirm an ADHD identity. For instance, algorithmically curated content on platforms like TikTok, aggregated under hashtags such as #ADHD, provides a constant stream of relatable symptom portrayals that can trigger self-recognition [16]. Simultaneously, dedicated forums like Reddit’s r/ADHD function as extensive peer-review systems, where users compare their experiences against crowdsourced diagnostic criteria and find validation through community consensus [24]. This immersive exposure to symptomology, while informative, may also heighten health anxiety, further motivating the search for a definitive label. Thus, where clinical diagnosis grants verification through medical authority, self-diagnosis achieves it through a confluence of algorithmic exposure, personal resonance, and peer-affirmed consensus [2].

Self-verification through both formal and informal pathways plays a central role in shaping self-image and internalized stigma. Self-image refers to an individual’s perception of their personal traits, competencies, and overall worth [25], while internalized stigma involves adopting negative societal beliefs about one’s condition [26], leading to diminished self-esteem and self-efficacy. For individuals with a clinical ADHD diagnosis, medical validation can strengthen self-image by reframing distressing experiences as manifestations of a neurobiological condition rather than personal failure, thus promoting a coherent and stable self-concept [27]. Likewise, self-diagnosed individuals often construct a revised identity through the lens of neurodivergence, gaining validation and support within online communities [24]. Although both groups engage in identity reconstruction, the perceived legitimacy and psychological security derived from the diagnosis process may differ by verification source.

At the same time, these verification processes expose individuals to internalized stigma. Clinically diagnosed individuals may internalize stereotypes associated with psychiatric labeling, such as assumptions of reduced competence or dependency, which can undermine autonomy and reinforce negative self-evaluations [28,29]. In contrast, self-diagnosed individuals frequently contend with stigma tied to perceived illegitimacy. The absence of professional validation can foster persistent self-doubt, imposter syndrome, and anxiety over the credibility of their condition [30]. These divergent sources and expressions of internalized stigma warrant empirical comparison to better understand their impact on self-concept. This leads to the following research question:

**RQ1**: How do self-image and internalized stigma differ between individuals with self-diagnosed and clinically diagnosed ADHD?

### Pathways to verification: Social and media validation

The fundamental drive to maintain a coherent self-concept, as posited by Self-Verification Theory, manifests through the active pursuit of external validation. This process operates through two primary channels distinguished by their source. The first, social validation, is defined as confirmation sought from or provided by interpersonal sources, such as family, friends, and partners, that affirms an individual’s ADHD-related experiences [9,31]. The second, media validation, encompasses confirmation sought from impersonal and parasocial sources that reflect the user’s experiences, including mass media portrayals and large-scale online communities where peer consensus functions as a form of impersonal confirmation [24].

The degree of reliance on these validation channels is hypothesized to differ systematically based on diagnostic status. A formal clinical diagnosis functions as a powerful, authoritative act of verification that may reduce an individual’s dependence on other sources for identity confirmation. In contrast, self-diagnosed individuals lack this formal anchor and are theoretically more dependent on continuous social and media feedback to construct and solidify their self-concept [30]. This hypothesized difference in behavior warrants empirical investigation, leading to the following research question:

**RQ2:** What are the differences in the prevalence and types of validation seeking (social and media) between self-diagnosed and clinically diagnosed individuals with ADHD?

Beyond establishing behavioral patterns, it is critical to ascertain the psychological import of validation seeking. The process is not neutral; affirming validation can bolster self-image by reframing personal struggles as manifestations of a legitimate condition, thereby mitigating self-blame and internalized stigma [27]. Self-image refers to an individual’s subjective evaluation of themselves, encompassing beliefs about personal worth, competence, and identity [32], while internalized stigma represents the process by which individuals accept and integrate negative societal beliefs or stereotypes about a stigmatized identity into their own self-concept [26]. Conversely, encountering skepticism or stigmatizing portrayals can erode self-image and intensify negative self-perceptions (Mueller and colleagues, 2012) [33]. To examine this direct relationship between the validation-seeking process and its psychological outcomes, the study asks:

**RQ3:** How are the types of validation seeking (social and media) associated with self-image and internalized stigma?

Finally, the psychological impact of validation seeking is likely contingent upon an individual’s diagnostic status. For clinically diagnosed individuals, a formal medical diagnosis serves as an authoritative anchor, potentially buffering them against the harm of negative feedback [34]. In contrast, the self-concept of self-diagnosed individuals is inherently more tenuous and thus more susceptible to external evaluation [1]. This differential vulnerability suggests that diagnosis status may function as a key moderator, altering the strength and nature of the relationship between validation seeking and its psychological outcomes. To investigate this proposed interactive effect, the study asks:

**RQ4:** Does an individual’s diagnosis status (self-diagnosed versus clinically diagnosed) moderate the relationship between the types of validation seeking (social and media) and their self-image and internalized stigma?

## Methods

### Data collection

We selected Reddit for this study due to its large, anonymous user base, which facilitates research on marginalized conditions [35]. We examined the r/adhd, a public community with 1.9 million members. Using the Pushshift API, we collected 452,026 posts made by 19,738 unique users between April 2009 and December 2023. Data collection complied with Reddit’s API terms of service and followed ethical guidelines for public data. Ethical approval was not required as the study did not involve interaction with human subjects and used publicly accessible data [36].

### Coding scheme and annotation process

We initiated the coding scheme development with a preliminary framework derived from established literature on self-verification theory [8], self-concept theory (Rogers, 1959) [37], and stigma literature [26]. Two authors then used this initial scheme to independently code a random sample of 200 posts, achieving a high inter-coder reliability (Cohen’s Kappa = 0.92). The authors resolved all disagreements through discussion, refining the codebook definitions and incorporating concrete, data-driven examples. This iterative process resulted in the final, validated codebook presented in Table 1.

**Table 1:** Coding scheme for key constructs.

Construct	Definition	Examples
**Diagnosis Type**	The diagnostic status as reported by the user, distinguishing between self-ascribed identity and formal medical validation.	
Self-Diagnosis	A self-ascribed identification with ADHD in the absence of a reported formal medical assessment.	“After trying to figure out what’s wrong with me, I’ve come to the conclusion that I have ADHD”; “I’m undiagnosed but suspecting things... I started to write down and read this subreddit.”
Clinical Diagnosis	A reported diagnosis of ADHD obtained through a formal medical process involving a healthcare professional.	“I was diagnosed a day ago... my doctor prescribed a new medicine”; “I got diagnosed yesterday after studying in college for a while.”
**Validation Seeking**	The active search for external information that confirms a user’s self-concept of having ADHD, consistent with self-verification theory [8].	
Social Validation	Confirmation sought from or provided by interpersonal sources (e.g., family, friends, partners) that affirms the user’s ADHD-related experiences.	“I described my symptoms to my mother, and she said my father had the exact same struggles”; “People say ‘I think you might have ADHD’ fairly frequently”
Media Validation	Confirmation sought from or provided by impersonal or parasocial sources (e.g., online communities, media portrayals) that reflect the user’s experiences.	“It wasn’t until I saw a TikTok compilation of ADHD symptoms that everything clicked; for the first time, I felt truly seen”; “Reading through posts on this subreddit confirmed that what I’ve been experiencing my whole life is real.”
**Self-Image**	The user’s evaluation of their own worth or capability in relation to their ADHD, based on self-concept theory (Rogers, 1959).	
Positive Self-Image	A positive evaluation of the self where ADHD is framed as a source of strength, creativity, or unique identity.	“Overall it’s great. ADHD gives me energy and makes me make people laugh”; “I feel like it’s a gift… limitless creativity, obsessive motivation.”
Negative Self-Image	A negative evaluation of the self where ADHD is framed as a source of failure, frustration, or personal inadequacy.	“ADHD completely destroyed my adolescence... I have come to despise the person my inaction has made me”; “I didn’t get the job... I feel like a piece of shit.”
**Internalized Stigma**	The endorsement and application of negative societal stereotypes about ADHD to oneself, resulting in feelings of shame or diminished self-worth [26].	“I feel ashamed for needing accommodations”; “I avoided treatment for years because of the source of shame.”

*Note.* Example are drawn from posts on the r/adhd subreddit and anonymized.

This codebook was then used to construct a prompt for large-scale annotation with GPT-4. The prompt instructed the model to classify each post as present (1) or absent (0) for each category. To validate the model’s performance, two authors and GPT-4 independently annotated a separate set of 500 posts, achieving strong inter-rater agreement (Cohen’s Kappa = 0.86). An analysis of the disagreements identified systematic model errors, and the prompt was subsequently updated with more precise instructions and edge-case examples. This refined prompt was used to annotate the full 4,000-post training dataset. A final reliability check on a new random sample of 200 posts confirmed that high accuracy was maintained (Cohen’s Kappa = 0.90).

### Supervised classification using natural language processing

We began data preprocessing by using regular expressions to remove rows containing deleted posts or URLs. Next, we processed the remaining text with the SpaCy library [38] to perform lemmatization, stop-word removal, and conversion to lowercase.

Following preprocessing, the text was tokenized using the RoBERTa tokenizer [39]. An optimized model effective at capturing contextual information. To address class imbalance, BorderlineSMOTE oversampling was applied [40]. Subsequently, the dataset was split into training (80%), validation (10%), and test (10%) sets using stratified sampling.

A pre-trained RoBERTa sequence classification model was fine-tuned for 25 epochs using the AdamW optimizer [41] with a small learning rate to ensure stability. The model was optimized to near-zero training loss to enhance generalization [0]. Performance was evaluated on the validation set after each epoch, and the checkpoint with the highest F1 score was selected for final evaluation. Table 2 presents the model’s performance on the test set. The trained classifier was then applied to the full unlabeled dataset to assign predicted classes and probabilities to each post.

**Table 2:** Classification performance.

Classification Task	Precision	Recall	F1 score	Accuracy
Self-Diagnosis	0.92	0.91	0.91	0.91
Clinical diagnosis	0.89	0.89	0.89	0.89
Positive Self-Image	0.95	0.95	0.95	0.95
Negative Self-Image	0.79	0.78	0.78	0.78
Social Validation	0.83	0.81	0.81	0.81
Media Validation	0.88	0.88	0.88	0.88
Internalized Stigma	0.79	0.79	0.79	0.79

*Note.* Self-Diagnosis: precision (0.86, 0.98), recall (0.98, 0.84), F1-score (0.92, 0.90) for Not Self-Diagnosis and Self-Diagnosis respectively. Clinical diagnosis: precision (0.94, 0.85), recall (0.83, 0.94), F1-score (0.88, 0.89) for Not Clinical diagnosis and Clinical diagnosis respectively. Professional Validation: precision (0.90, 0.86), recall (0.85, 0.90), F1-score (0.87, 0.88) for Not Professional Validation and Professional Validation respectively. Social Validation: precision (0.92, 0.75), recall (0.68, 0.94), F1-score (0.78, 0.83) for Not Social Validation and Social Validation respectively. Internalized Stigma: precision (0.84, 0.75), recall (0.71, 0.87), F1-score (0.77, 0.80) for Not Internalized Stigma and Internalized Stigma respectively.

### Data analysis

The initial dataset included 435,048 posts from 188,318 unique users on the r/ADHD subreddit. Two analytical subsets were created based on users’ self-reported diagnosis status: posts from users with self-diagnosed ADHD (n = 23,507) and posts from users with clinical diagnoses (n = 188,318). The final analysis included 211,825 posts from 15,432 unique users, focusing exclusively on posts where diagnosis status could be clearly identified.

All statistical analyses were conducted in R. To mitigate the risk of Type I error from multiple comparisons, we applied the Benjamini-Hochberg procedure to control the False Discovery Rate (FDR) at a 5% threshold [42].

To examine RQ1 and RQ2, chi-squared tests were used. These tests compared he prevalence of positive/negative self-image, internalized stigma, and social/media validation seeking between the self-diagnosed and clinically diagnosed groups. The assumptions for this approach were satisfied, as all expected cell frequencies were greater than five.

Prior to modeling, all categorical predictors were dummy-coded, and an assessment of Variance Inflation Factors (VIFs < 1.2) confirmed that multicollinearity was not a concern. To analyze the relationships in RQ3 and RQ4, we adopted a modeling strategy appropriate for the data’s hierarchical structure. Because multiple posts were nested within individual users, standard regression methods would violate the assumption of independent observations. We therefore employed generalized linear mixed-effects models (GLMMs), which account for this hierarchical structure by incorporating a random intercept for each user [43].

The analysis was conducted sequentially. First, to address RQ3, baseline models were specified to examine the main effects of social and media validation seeking on each of the three outcomes. Subsequently, to test the moderation hypothesis in RQ4, these models were expanded to include diagnosis status as a predictor, along with its interaction with each validation type. A significant interaction term indicated that diagnosis status moderated the relationship between validation seeking and the outcome; we confirmed the significance of this moderation by using likelihood ratio tests to evaluate the improvement in overall model fit (see Table 4).

**Table 4:** *Summary of hierarchical logistic mixed-effects m*odels.

Predictor	Negative Self-Image	Positive Self-Image	Internalized Stigma
	*b* (SE)	*b* (SE)	*b* (SE)
**Fixed Effects**			
Intercept	−0.88 (0.01)***	−9.23 (0.04)***	−0.57 (0.01)***
Social Validation	1.17 (0.01)***	1.15 (0.03)***	1.24 (0.01)***
Media Validation	0.00 (0.03)	0.14 (0.08)	0.04 (0.03)
Diagnosis Status	1.70 (0.02)***	0.12 (0.07)	1.51 (0.02)***
Date (Scaled)	0.08 (0.01)***	−0.01 (0.02)	0.10 (0.01)***
*Interactions*			
Social Validation × Diagnosis Status	−0.97 (0.03)***	−0.42 (0.09)***	−0.83 (0.03)***
Media Validation × Diagnosis Status	−0.31 (0.07)***	−0.51 (0.21)*	−0.18 (0.07)*
**Random Effects**			
Intercept (User) Variance	0.36	158.56	0.33
**Model Fit**			
Model Comparison (vs. Main Effects) χ² (2)	894.92***	23.32***	577.13***
*N* (observations)	200,770	200,770	200,770
*N* (users)	130,810	130,810	130,810

***Note.*** *b* = unstandardized coefficient from the logistic mixed-effects model (log-odds); SE = Standard Error. Date was included as a scaled control variable. Diagnosis Status was coded with Clinical Diagnosis as the reference group (0) and Self-Diagnosis as the comparison group (1). The Model Comparison statistic is the Likelihood Ratio Test χ² value comparing the final model with interactions against a main-effects-only model. *p < .05. *p < .01. **p < .001.

## Result

Initial analyses for RQ1 and RQ2 were conducted at the user level (N = 88,591), where each user was classified based on having at least one post exhibiting the variable of interest. These chi-squared tests of association utilized Cramer’s *V* to assess effect size, where values approaching 0.1 indicate a small effect and those nearing 0.3 suggest a medium effect.

RQ1 examined differences in validation seeking. Self-diagnosed individuals were significantly more likely than their clinically diagnosed counterparts to seek social validation (χ²(1) = 3,550.65, *p* < .001), and media validation (χ²(1) = 419.65, *p* < .001). The effect size for the former was small (Cramer’s *V* = 0.13), while the latter was negligible (Cramer’s *V* = 0.05).

RQ2 focused on differences in self-image. Compared to the clinically diagnosed group, self-diagnosed individuals were significantly more likely to express a negative self-image (χ²(1) = 9,077.43, *p* < .001) and internalized stigma (χ²(1) = 7,479.43, *p* < .001). The corresponding effect sizes were small-to-moderate (Cramer’s *V* = 0.21 and *V* = 0.19, respectively). No significant group difference emerged for positive self-image (χ²(1) = 1.72, *p* = .190). A summary of these findings is available in Table 3.

**Table 3:** Chi-square test results for RQ1 and RQ2.

Variable	Self-Diagnosed (%)	Professional Diagnosed (%)	*χ²*(1)	p	Effect Size
*Positive Self Image*	10.71	11.00	1.72	0.190	0.00
*Negative Self Image*	69.77	37.23	9,077.43	<.001	0.21
*Internalized Stigma*	73.98	44.00	7,479.43	<.001	0.19
*Social Validation*	44.69	26.07	3,550.65	<.001	0.13
*Media Validation*	5.62	3.06	419.65	<.001	0.05

*Note.* Analyses for this table were conducted at the user level (*N* = 88,591), where each user was classified based on having at least one post present for the given variable. Effect sizes are reported as Cramer’s V.

To address RQ3 and RQ4, while also accounting for potential temporal trends in our 14-year dataset, we conducted a series of mixed-effects logistic regression models on the full dataset of 200,770 posts from 130,810 unique users (see Table 4). We included the post’s date as a scaled control variable in all models. This time variable emerged as a significant positive predictor of both negative self-image (*b* = 0.08, *p* < .001) and internalized stigma (*b* = 0.10, *p* < .001), suggesting a gradual increase in the discussion of these negative experiences over the years.

After controlling for time, the main effects model (RQ3) revealed that social validation remained a strong, significant predictor, positively associated with expressing a negative self-image (*b* = 1.17, *p* < .001), a positive self-image (*b* = 1.15, *p* < .001), and internalized stigma (*b* = 1.24, *p* < .001). Media validation, however, was not significantly associated with any outcome.

**Fig 1:**
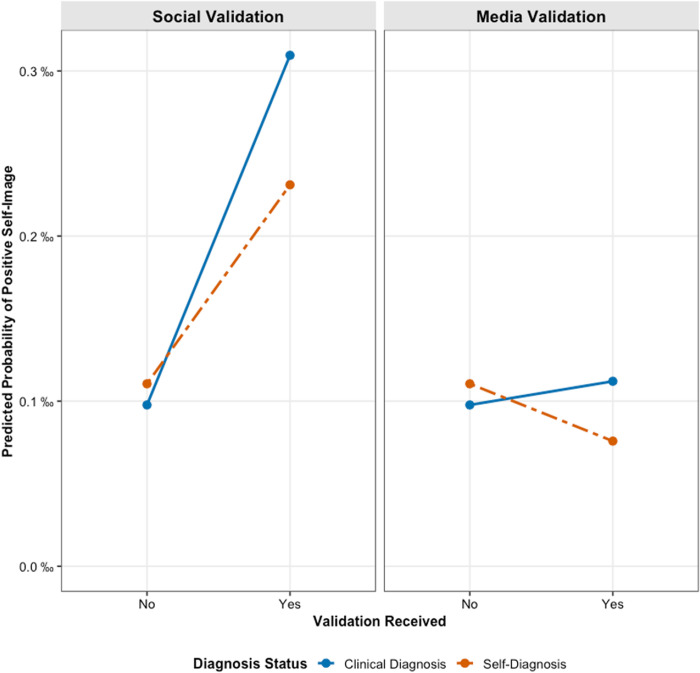
Effects of social and media validation on positive self-image by diagnosis status.

**Fig 2:**
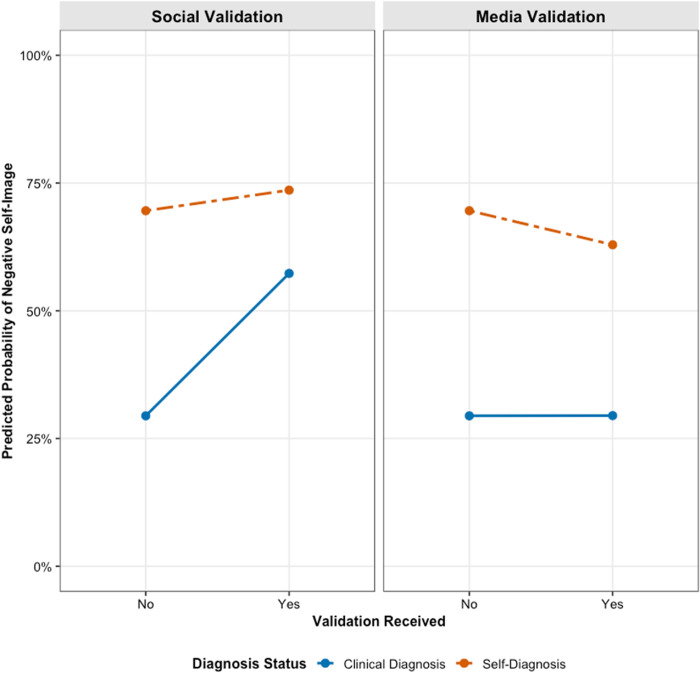
Effects of social and media validation on negative self-image by diagnosis status.

**Fig 3:**
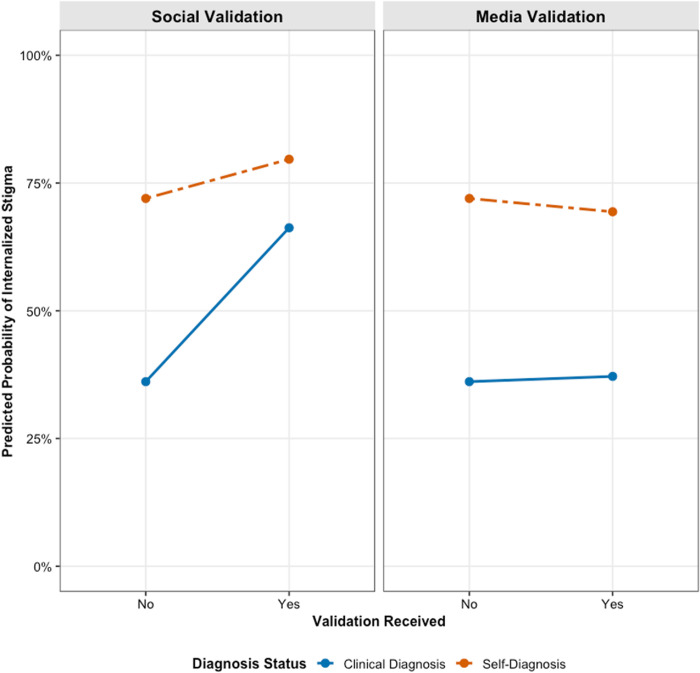
Effects of social and media validation on internalized stigma by diagnosis status.

For RQ4, we introduced interaction terms to test whether diagnosis status moderated these relationships. Adding the interactions significantly improved model fit for negative self-image (χ²(2) = 894.92, *p* < .001), positive self-image (χ²(2) = 23.32, *p* < .001), and internalized stigma (χ²(2) = 577.13, *p* < .001). As illustrated in Figs 1–3, diagnosis status consistently moderated the effect of social validation. The positive associations between social validation and negative self-image, positive self-image, and internalized stigma were all significantly weaker for self-diagnosed individuals compared to clinically diagnosed individuals (*b* = −0.97, *p* < .001; *b* = −0.42, *p* < .001; and *b* = −0.83, *p* < .001, respectively). The influence of media validation was also significantly moderated, although the interaction effects were smaller in magnitude. The positive associations between media validation and negative self-image (*b* = –0.31, *p* < .001), positive self-image (*b* = –0.51, *p* < .05), and internalized stigma (*b* = –0.18, *p* < .05) were significantly weaker for self-diagnosed individuals compared to those with a clinical diagnosis.

## Discussion

This study investigated the complex relationships between ADHD diagnosis status, online validation-seeking, and self-perception. Our findings reveal three key insights: first, self-diagnosed individuals are more likely to seek validation online, a behavior driven by systemic barriers to formal diagnosis; second, social validation has a paradoxical effect, being associated with both positive and negative self-perceptions; and third, a clinical diagnosis functions as a crucial moderator, shaping how validation influences an individual’s self-image and internalized stigma.

### The drive for validation: Self-verification and barriers to diagnosis

Our finding that self-diagnosed individuals more actively seek online validation aligns with Self-Verification Theory [8]. The theory posits that individuals are motivated to obtain feedback that confirms their pre-existing self-concepts. For individuals who self-identify as having ADHD but lack formal recognition, online platforms serve as a crucial arena for this identity verification process. This behavior reflects not merely a search for support but a cognitive drive to solidify a tentative aspect of their identity.

This behavior is further contextualized by the significant barriers to formal diagnosis, which include prohibitive costs, fear of dismissal by healthcare providers, and systemic inequities [44,45]. Consequently, when individuals perceive the medical establishment as inaccessible or invalidating, they often turn to online peer communities. Here, they can share experiences and receive feedback that confirms their self-view without the gatekeeping and power dynamics inherent in the clinical setting [46,47].

### The paradox of social validation and its impact on self-perception

Our analysis of main effects revealed a critical distinction between validation from media and social sources. Media validation showed no significant main effect on self-image or internalized stigma, suggesting that passive consumption of media may be insufficient to alter deeply held self-perceptions [48]. In stark contrast, social validation emerged as a powerful yet paradoxical predictor of self-perception. It was strongly and positively associated with expressing a negative self-image and internalized stigma, while simultaneously being linked to a positive self-image.

Self-Verification Theory offers a compelling explanation for this paradox by positing that individuals seek to confirm their existing self-concept [8], encompassing not only valued traits but also stigmatized ones. From this perspective, when social validation affirms an individual’s ADHD-related experiences, it can provide a coherent explanatory framework that enhances self-esteem [24]. However, this same process simultaneously reinforces negative self-perceptions by affirming the very struggles and perceived deficits that contribute to internalized stigma (Bussing & Mehta, 2013; Foster & Ellis, 2024) [49]. Therefore, social validation functions as a dual-edged process in which the confirmatory mechanism that builds a positive identity also entrenches its most limiting and stigmatized elements.

### The moderating role of diagnosis status in shaping self-image and stigma

Our moderation analyses reveal that a clinical diagnosis fundamentally alters the effectiveness of validation. Specifically, social validation has significantly stronger associations with psychological outcomes among clinically diagnosed individuals. This heightened responsiveness aligns with Self-Verification Theory, which posits that individuals with established self-concepts derive greater psychological significance from relevant feedback [8]. A clinical diagnosis appears to provide the confidence needed to seek and internalize this social feedback, thereby facilitating a more meaningful self-verification process. However, the same formal label can also function as a stigma marker [50], inviting the invalidating commentary that explains the paradoxical link between social validation and negative self-perception.

Conversely, self-diagnosed individuals experienced attenuated effects from validation despite seeking it more frequently. This finding highlights the challenge of self-verification without an established identity foundation, a paradox consistent with the theory’s predictions regarding self-concept uncertainty [8]. The identity ambiguity inherent in self-diagnosis means that social validation cannot definitively confirm a mental health status. As a result, individuals may become trapped in a cycle of seeking validation that yields diminishing psychological returns. The absence of professional verification thus undermines the certainty required for the self-verification process to be effective.

Finally, the moderation effects of media validation were significantly attenuated compared to those of social validation across all outcomes. This distinction stems from the fundamental difference between the sources: social validation is direct and reciprocal, whereas media validation is parasocial and unidirectional [51]. Although media content can foster a sense of recognition, it lacks the personalized feedback necessary for meaningful identity confirmation [52]. Thus, even when clinical diagnosis heightens individuals’ attention to media, the impersonal nature these sources may shape self-perception less than direct interpersonal feedback.

### Practical implications

These findings extend self-verification theory by demonstrating how professional verification creates a hierarchical structure that moderates social verification effectiveness. Traditional applications focus primarily on social feedback as the mechanism for self-concept confirmation. However, our results suggest clinical diagnosis establishes the foundational certainty necessary for meaningful social verification [46]. This hierarchical model has significant clinical implications: the diagnostic process emerges not merely as assessment but as intervention that enables effective engagement with social support systems. For self-diagnosed individuals, pursuing professional confirmation becomes essential for establishing the self-concept stability required for beneficial self-verification in social contexts.

Online communities and social media shape how individuals view themselves. These platforms should share accurate information that supports a positive self-image and reduces stigma. Content creators and moderators can help by combining personal stories with reliable educational material. This mix can create a supportive space that validates experiences without reinforcing negative stereotypes [47]. Collaboration with mental health organizations is essential to ensure the dissemination of evidence-based content, which can strengthen social support networks and promote positive mental health outcomes for the ADHD community.

## Limitations and future studies

This study’s findings should be interpreted in light of several limitations. First, the exclusive reliance on data from the r/ADHD subreddit introduces a considerable risk of selection bias. The user base of this online forum may not be representative of the broader ADHD population, as participants are self-selected and likely possess higher digital literacy and a greater propensity for seeking online community support. This sampling constraint is amplified by the well-documented demographic patterns of the platform, which may not reflect the full diversity of the global population affected by ADHD. Consequently, the generalizability of our results is necessarily limited.

Second, the study is constrained by its methodological design. Our dependence on Natural Language Processing, while validated by human coders, may not fully capture linguistic nuances such as sarcasm or irony, potentially impacting the accuracy of data classification. Furthermore, the binary operationalization of complex psychological constructs like self-image and internalized stigma, though necessary for analytical reliability at scale, simplifies their multidimensional nature by measuring presence rather than intensity. Finally, the study’s cross-sectional design illuminates statistical associations but precludes the establishment of causal relationships. It is therefore unable to elucidate the temporal dynamics between validation-seeking behaviors and changes in self-perception over time.

Future research should address these limitations by using multiple methods. Qualitative research could offer deeper insights into how validation affects self-perception and stigma. A crucial area for subsequent investigation is the role of healthcare providers. Research examining how clinicians respond to self-diagnosed individuals and how their communication styles affect patient outcomes could inform the development of enhanced training programs, thereby improving support and understanding for the broader ADHD community.

## Conclusions

This study analyzed validation-seeking behaviors among self-diagnosed and clinically diagnosed individuals in the r/ADHD community. We found that self-diagnosed individuals sought significantly more social and media validation. However, this social validation had a paradoxical effect: it enhanced positive self-concept while also reinforcing internalized stigma. Furthermore, diagnosis status moderated this effect, as clinically diagnosed individuals showed stronger associations between social validation and both outcomes. Together, these findings illuminate distinct pathways of ADHD identity formation and call for targeted interventions. Healthcare providers could integrate discussions of online community engagement into diagnostic practice, while digital platforms could curate content that validates experiences without reinforcing stigma. Understanding these social and media validation dynamics becomes essential for supporting neurodivergent individuals who navigate identity confirmation across both clinical and digital environments.
